# HIV Prevalence and Risks Associated with HIV Infection among Transgender Individuals in Cambodia

**DOI:** 10.1371/journal.pone.0152906

**Published:** 2016-04-12

**Authors:** Amy Weissman, Song Ngak, Chhim Srean, Neth Sansothy, Stephen Mills, Laurent Ferradini

**Affiliations:** 1 FHI 360, Phnom Penh, Cambodia; 2 FHI 360, Bangkok, Thailand; 3 National Center for HIV/AIDS, Dermatology and Sexually Transmitted Diseases, (NCHADS) and University of Health Science, Phnom Penh, Cambodia; University of Missouri-Kansas City, UNITED STATES

## Abstract

**Introduction:**

Recognizing transgender individuals have a high risk of HIV acquisition, and to inform policies and programming, we conducted an HIV prevalence and risk behaviors survey among transgender individuals in Cambodia.

**Methods:**

Cross-sectional survey using a respondent driven sampling method with self-administered audio-computer assisted interviews. HIV testing was performed prior to the questionnaire with results available immediately after. Eligible participants were ≥18 years, identified as male at birth and self-identified/expressed as a different gender, and reported having sex with at least one male partner in past year. From six major urban centers of Cambodia, 891 transgender individuals were recruited.

**Results:**

The majority of the 891 participants self-identified as third gender or female (94.5%), were young (median age 23, IQR [20–27]), had secondary education or higher (80.5%), not married (89.7%), and employed (90.2%). The majority had first sex before 18 years (66.8%), with a male (79.9%), 37.9% having been paid or paying for this first sex. The rate of HIV positivity among participants was found to be 4.15%. Consistent condom use with male and female partners was low with all partner types, but particularly low with male partners when paying for sex (20.3%). The majority of participants reported having experienced discrimination in their lifetime (54.8%) and 30.3% had been assaulted. Multivariate analysis revealed that older age (adjusted OR = 14.73 [4.20, 51.67] for age 35–44 and adjusted OR = 7.63 [2.55, 22.81] for age 30–34), only having a primary school education or no schooling at all (adjusted OR = 2.62 [1.18, 5.80], being a resident of Siem Reap (adjusted OR = 7.44 [2.37,23.29], receiving payment at first sex (adjusted OR = 2.26 [1.00, 5.11], having sex during/after using drugs (adjusted OR = 2.90 [1.09,7.73]), inconsistent condom use during last anal sex (adjusted OR = 3.84 [1.58, 9.33]), and reporting low self-esteem (adjusted OR = 3.25 [1.35,7.85]) were independently associated with HIV infection.

**Conclusions:**

This study confirms transgender individuals as one of the highest-risk groups for HIV infection in Cambodia. It suggests the need for programmatic strategies that mitigate identified associated risks and facilitate access to HIV care for this population.

## Introduction

Transgender women (sometimes referred to as male-to-female transgender) have a high risk of HIV transmission globally [1–3]. According to a 2012 systematic review and meta-analysis, transgender women carry a very high burden of HIV with a pooled prevalence of 19.1% across 15 countries. Three of these countries with a high HIV prevalence among transgender women, Thailand (12.15%) Indonesia (26.1%), and Vietnam (6.67%), have concentrated epidemics similar to that of Cambodia [1]. This review suggests that transgender women are a high priority population for intensive combination prevention interventions and access to ARV treatment.

Although transgender women share similar risk factors with men who have sex with men (MSM), like unprotected receptive anal intercourse, this population faces additional and distinct risks and sexual health needs at multiple levels: individual (including depression, substance use, illicit hormone and silicon injections), community (such as lack of social support and social exclusion) and from the socio-cultural environment (for example, limited economic opportunities, limited access to services, and stigma and discrimination) [1, 2, 4].

Transgender persons in Cambodia also have an elevated risk of HIV infection. According to a 2005 study of sexually transmitted infections (STIs), transgender persons (N = 193) in three sites had an HIV prevalence of 9.8% [5]. Another study (2010) conducted among high risk men in seven provinces, found transgender participants (N = 379) had a prevalence of 2.6% [6].

According to the 2007 Behavioral Sentinel Surveillance, transgender persons in Cambodia also have high rates of risk behaviors, and higher than those of MSM. The majority of transgender persons reported ever having sold sex (60% vs. 36% of MSM), and among all who had sold sex, transgender persons reported first selling sex at an earlier age [7]. Further, the first sexual partner of transgender individuals was more often a man (93%), while for MSM it was more often a woman (56%). Transgender persons also reported less consistent condom use with all sexual partners [7]. In Cambodia, transgender individuals tend to identify clients in entertainment venues, such as specific bars and clubs considered transgender persons’ hotspots, and on the street. Sex work among transgender individuals in Cambodia falls within the legal context of sex work, particularly the Law on the Suppression of Human Trafficking and Sexual Exploitation, 2008.

Although transgender persons have been recognized as having higher prevalence rates and risk behaviors than other populations in Cambodia, including MSM, this population was programmatically reached simultaneously with MSM, with no distinct behavior change communications or services. This means that transgender individuals’ specific needs were neither well understood nor addressed. In keeping with WHO’s recommendation to “avoid conflating MSM and transgender [8]”, Cambodia’s National Center for HIV/AIDS, Dermatology, and Sexually Transmitted Diseases (NCHADS) produced in 2013 a Standard Operating Procedure (Boosted Continuum of Prevention, Care and Treatment (B-CoPCT) SOP) in which transgender persons were identified distinctly as one of four key populations [9]. In support of this SOP, and to inform appropriate targeting and service delivery for transgender persons in Cambodia, we conducted an integrated biological behavioral and surveillance study (IBBSS) including HIV prevalence evaluation.

## Methods

### Respondent Driven Sampling (RDS) and population studied

Using Respondent Driven Sampling (RDS) [10], participants were recruited across six Cambodian cities (Phnom Penh, Battambang, Banteay Mean Chhey, Kampong Cham, Siem Reap and Sihanoukville), considered priority sites for HIV programming, and nationally representative of the country’s major urban areas. In each city, two to three ‘seeds’ known to have a strong social network, with 10 or more transgender friends, were identified by NGO Community or Peer Facilitators who conduct HIV prevention outreach among transgender individuals. These seeds were referred to the study team for selection. Recruitment was stratified by location. The target was as follows: 500 individuals in Phnom Penh, 200 in Banteay Meanchey (including 50 in Serey Saophoan & 150 in Poipet), 150 in Battambang, 150 in Siem Reap; and 100 in Kampong Cham and Sihanoukville. Recruitment in each site stopped when the target was reached. To reach saturation, each seed was expected to expand to five or six recruitment waves in each city.

Study participants were eligible if aged ≥18 years, identified as male at birth and self-identifying and/or expressing themselves as a different gender (pre- and post-operative individuals included), and reporting having sex with at least one male partner in the past year. While considering only male to female/third gender transgender individuals, this definition is similar to the UNAIDS definition of transgender: ‘a transgender person has a gender identity that is different from his or her sex at birth’ [11] either from male to female (female appearance) or from female to male (male appearance). Each seed was given two coupons and asked to refer two transgender individuals. Referred individuals were again screened by field interviewers, ensuring their eligibility and desire to participate. For a successful referral, seeds were given a phone card valued at 1 US dollar (USD), and received a maximum of 2 phone cards valued at 2USD for two successful referrals. Participants were given phone cards valued at 2USD to compensate them for their time. Seeds were included as study participants and included in the data analysis.

### HIV testing

HIV testing was conducted prior to the interview using the national algorithm for HIV prevalence surveillance, according to the national guidelines for HIV testing in surveillance, which recommend a combination of two assays be used for all sentinel groups, regardless of HIV prevalence [12]. Rapid testing was performed on site when the participants were interviewed and all specimens were tested using both Determine^™^ and Stat-Pak^™^ assays with Determine^™^ considered as the first test in the algorithm. Specimens reactive with Determine^™^ and non-reactive with Stat-Pak^™^ were considered HIV negative. For specimen non-reactive with Determine^™^ and reactive by Stat-Pak^™^, both tests were repeated. Only if two or more results were reactive were the results considered positive. A tie-breaker test was not conducted as part of the study because such tests are only allowed to be done in VCT clinics. However, participants with a reactive test were given referrals for a confirmatory test at an HIV counseling and testing site. Post-test counseling and other referrals were provided to all participants after completion of the self-administered questionnaire.

For quality control, additional blood was collected from 20% of participants following enrollment order in each city. Quality control testing entailed testing the dried blood spots (DBS) with one enzyme-linked immunosorbent assay (ELISA), e.g., Vironostika^™^ HIV Uni-Form II Plus O^®^ (Organon Teknika), and if positive, the sample was retested with Murex^™^ HIV-1.2.O (Abbott Diagnostics). Quality control was conducted in the NCHADS laboratory and results were compared with those of the rapid HIV testing conducted in the field.

## Data collection and management

Quantitative data and biological samples were gathered in existing drop-in-centers run by local NGOs from 10 August to 15 September 2012. Quantitative data were gathered via self-administered interviews using an Audio-Computer Assisted Survey Instrument (ACASI) on a laptop or tablet. The questionnaire was divided into eight sections: socio-demographic characteristics; access to/participation in HIV prevention programs; transgender identity and experiences; STI and HIV testing; sexual partners and sexual history; alcohol and drug use; HIV prevention knowledge; and stigma, violence, and social support. Response options for the gender identity question were: male, female or third gender. This reflects the term “Kathoey” historically used in Cambodia to refer to both MSM and transgender individuals. It means third gender (or third sex), neither male nor female. Because gender identity is a fluid concept for people in Southeast Asia [13, 14], people who self-identified as male but presented as female were also considered transgender persons. Questions related to socio-demographic characteristics, STI and HIV testing, sexual partner and sexual history, and HIV prevention knowledge were adapted from global BSS guidelines [15]. Questions related to drug use included options for substances commonly used in Cambodia, including Yama, methamphetamine pills, and ICE, a methamphetamine powder, which is usually smoked or injected. Because it is less expensive than heroin, ICE sometimes serves as a heroin replacement. Questions related to stigma and discrimination, violence and social support were adapted from the People Living with HIV Stigma Index, and include a single measure of perceived self-esteem [16]. All questions were pre-tested with members of the study population. Questions were programmed into a tablet interface with an optional audio recording of all questions and corresponding choices for participants who had difficulty reading. As a self-administered questionnaire, participants selected their own answers, ensuring the anonymity of their responses.

Verbal informed consent was given by all participants to maintain their confidentiality and anonymity. Rather than names, study identification numbers were used on the consent forms, which were signed by the interviewers and the team supervisor who served as a witness, once consent was given. The study protocol, including this consent procedure, was reviewed and approved by the Cambodian National Ethics Committee on 30 July 2012 and the Protection of Human Subjects Committee (PHSC) of FHI 360 on 18 June 2012.

## Statistical analysis

STATA (Version 11.0 for windows, Stata Corp, TX, U.S.) was used to conduct data analysis. Univariate and multivariate logistic regression analysis were performed to analyze the risks associated with HIV infection. Variables with P ≤ 0.05 (two-tailed) in univariate analyses were all included in multivariate logistic regression. Backward one by one elimination of the variable that has highest p-value (>0.05) was used to identify remaining significant (P < 0.05) independent risk factors associated with HIV infection.

## Results

Using the RDS approach, 22 seeds were selected, 18 of which (81.8%) recruited participants. Of the 991 individuals with a coupon screened, 869 (87.7%) were eligible for the study, including the 22 initial seeds, a total of 891 participants were enrolled in the study to be tested for HIV and to answer the self-administered questionnaire using ACASI. To reach the required sample size in each province, the number of waves varied, with the most waves in Phnom Penh (n = 19), then in Siem Reap (n = 14), and Banteay Meanchey (n = 9).

### Socio-demographic characteristics

The 891 participants had a median age of 23 years (IQR: 20–27), with nearly two thirds (59.1%) below 25 years (Table 1). The majority self-identified as third gender (n = 606, 68.0%), followed by female (n = 236, 26.5%), and male (n = 49, 5.5%). Approximately 80% (n = 715) completed secondary school (both lower and higher), 43.4% (n = 387) were neither married nor living with any male partner, and 40.4% (n = 360) were not married but living with a male lover. Further, 90.2% (n = 804) of participants were employed mainly as beautician or hairdresser (n = 262, 29.4%), yet 50.2% (447 of 891) reported that their income did not meet their monthly expenses (data not shown). Among study participants, 42.2% (376 of 891) reported using hormones or having had sex re-assignment surgery to change their physical appearance (data not shown). Thus, study participants were in general young, educated, not married, and employed.

**Table 1:** Socio-Demographic Characteristics.

(n = 891)	n (%)
**Age** (years, Median [IQR])	23 [20–27]
*<25*	*527 (59*.*1)*
*25–29*	*223 (25)*
*30–34*	*89 (10*.*0)*
*35–44*	*39 (4*.*4)*
*> = 45*	*13 (0*.*1)*
**Gender identity**	
Third sex	606 (68.0)
Female	236 (26.5)
Male	49 (5.5)
**Education** (n = 870)	
*University & higher*	*217 (24*.*9)*
*Secondary*	*276 (31*.*7)*
*Lower secondary*	*222 (25*.*5)*
Primary level	126 (14.5)
Never attended school	29 (3.3)
**Current Residence**	
Phnom Penh	313 (35.1)
Battambang	145 (16.3)
Banteay Meanchey	118 (13.2)
Siem Reap	114 (12.8)
Kampong Cham	99 (11.1)
Preah Sihanouk	63 (7.1)
Other	39 (4.4)
**Marital status and relationship**	
Not married and not living with any partner	387 (43.4)
Not married, but living with male lover	360 (40.4)
Not married, but living with female sweetheart	53 (5.9)
Married and living together	48 (5.4)
Married but not living together	22 (2.5)
Widowed, divorced or separated	21 (2.4)
**Occupation**	
Teacher, office worker, government, NGO staff, student	171 (19.2)
Restaurant or café worker, street vendor, store seller	202 (22.7)
Beautician/Hairdresser	262 (29.4)
Sex worker	33 (3.7)
Farmer, Taxi/Tuk tuk, private driver	25 (2.8)
Factory worker	71 (7.9)
Other	40 (4.5)
Unemployed	87 (9.8)
**Alcohol use in the past 3 months** (n = 873)	
Never	285 (32.6)
Ever drink alcohol	470 (53.8)
Daily drink alcohol	118 (13.5)
**Drug use** (n = 870)	
Ever used drugs	188 (21.6)
Injecting drugs in the past 12 months	81 (9.3)
**Type of drug used in the past 3 months (n = 119)**	
Used Heroin	93 (78.2)
Used Yama	101 (94.9)
Used ICE and/or Amphetamines	64 (53.8)

Approximately half (470 of 873, 53.8%) of participants reported drinking alcohol in the past 3 months, and 13.5% (118 of 873) reported drinking daily (Table 1). Fewer participants reported ever having used drugs in their lifetime (188 of 870, 21.6%), including 9.3% (81 of 870) who reported ever injecting drugs in the past 12 months. Among those reporting drug use in the past three months (n = 119), 78.2% (n = 93) used Heroin, 94.9% (n = 101) used Yama and 53.8% used ICE (Table 1).

### Sexual behavior and history

Nearly all participants (874 of 891, 98.1%) reported having their first sexual experience before the age of 25, with 66.8% (595 of 891) at or before 18 years of age, and 18.1% (161 of 891) before 16 years of age (Table 2). Participants reported that their first sexual partner was most commonly a friend/neighbor (340 of 876, 38.8%), a sweetheart/boyfriend (312 of 876, 35.6%), or a stranger (187 of 876, 21.3%). The gender of this first sexual partner was most frequently male (708 of 886, 79.9%), and then transgender (105 of 886, 11.9%) or female (73 of 886, 8.2%). More than one-third (332 of 877, 37.9%) of participants had paid or were paid for their first sexual experience (Table 2), with more participants (294 of 891, 32.9%) reporting having received payment (data not shown). Payment for first sex was more common when having sex with a male (31.2%) than with a transgender individual (21.9%) or a female (13.1%) (data not shown). Forty one percent (366 of 891) of participants reported having commercial sexual partners (paid or paying) during the past 6 months (Table 2), among them 35.5% (130 of 366) had sex with more than 10 partners. Nearly one-third (261 of 857, 30.5%) of participants reported having sex with a female in their lifetime, 19.3% (165 of 857) during the past 6 months, with 9.7% (83 of 857) having been paid for that sex. Among those ever having sex with a female in their lifetime, only 18.8% self-reported as male, while 80.4% self-reported as female or a transgender individual. Similarly, among those having paid for sex with a female in the past 6 months, only 21.2% self-reported as male, while 88.7% self-reported as female or a transgender individual (data not shown). The majority (763 of 891, 85.6%) of participants reported having had anal sex with a male partner in their lifetime, 76.3% (680 of 891) in the past six months and 39.1% (348 of 891) having been paid for anal sex in the past six months (Table 2).

**Table 2:** Sexual Behavior and History.

	n (%)
**Age at first sex (years)** (n = 891, median [IQR])	18 [16–19]
<16	161 (18.1)
16–18	434 (48.7)
19–20	181 (20.3)
21–25	98 (11.0)
> 25	17 (1.9)
**Partner type at first sex** (n = 876)	
Friend/neighbor	340 (38.8)
Sweetheart/Boyfriend	312 (35.6)
Stranger	187(21.3)
Spouse	21 (2.4)
Family/relative	16 (1.8)
**Gender of first sexual partner** (n = 886)	
Male	708 (79.9)
Female	73 (8.2)
Transgender	105 (11.9)
**Payment at first sex** (n = 877)	
None	545 (62.1)
Was paid and/or paid	332 (37.9)
**Number of Commercial sexual partners during past 6 months** (n = 891, median [IQR])	7 [3–18]
None	525 (58.9)
1–5	164 (18.4)
6–10	72 (8.1)
>10	130 (14.6)
**Sex with women** (n = 857)	
Ever had sex with women	261 (30.5)
Had sex with any women during the past 6 months	165 (19.3)
Got paid for sex	83 (9.7)
**Sex with man** (n = 891)	
Ever had anal sex with man	763 (85.6)
Had anal sex with any man during the past 6 months	680 (76.3)
Got paid for sex in the past 6 months	348 (39.1)
**Condom use during the last anal sex with a man** (n = 763)	
Yes	640 (83.9)
No	123 (16.1)
**Sexual activity among those using alcohol in the past 3 months** (n = 561)	
Anal/vaginal sex after drinking alcohol	313 (55.8)
**Sexual activity among those using drug in the past 3 months**	
during/after using heroin (n = 93)	21 (22.6)
during/after using Yama (n = 101)	22 (21.8)
during/after using ICE and/or Amphetamine (n = 64)	40 (62.5)

**Fig 1:**
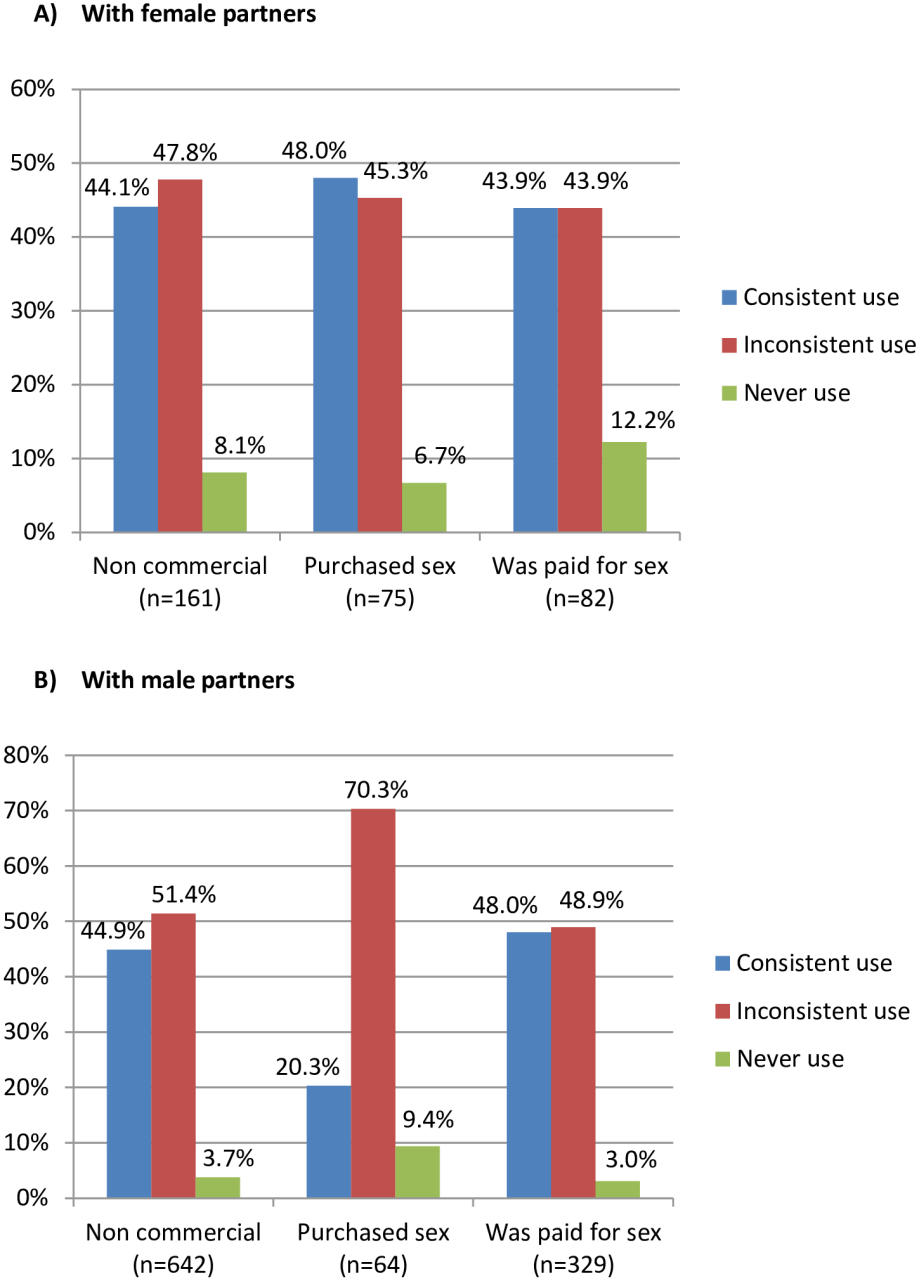
A. Condom use with partners during past 6 months by sex transaction type, with female partners. B. Condom use with partners during past 6 months by sex transaction type, with male partners.

Consistent condom use with female partners was overall low, never reaching above 50% for non-commercial or transactional sex during the past 6 months (Fig 1A). Overall condom use during last anal sex with a man was reportedly very high (640 of 763, 83.9%) (Table 2). However, consistent condom use with male partners was only 20.3% when paying for sex (compared to 48% with a female partner, p<0.001), and 48.0% when being paid for sex (compared to 43.4%, p = 0.5) during the past 6 months (Fig 1B). Among those who had inconsistent condom use at last anal sex, the majority self-identified as third gender (77.2%), with 17.1% self-identifying as female, and 5.7% as male (data not shown).

Among those who reported drinking alcohol and having had sexual activity during the past 3 months (n = 561), more than half (313 of 561, 55.8%) mentioned having sex after drinking alcohol (Table 2). During the past 3 months, having sex was more frequent during/after using ICE and/or amphetamines (40 of 64, 62.5%), than heroin (21 of 93, 22.6%) or Yama (22 of 101, 21.8%) (Table 2).

### Risk Perception, Stigma and Discrimination, and Violence

More than half (437 of 785, 55.7%) of participants reported feeling likely or very likely to be at risk of acquiring HIV (Table 3). The majority (488 of 891, 54.8%) also reported having experienced discrimination related to their transgender identity in their lifetime, and 30.3% (267 of 880) reported having been raped and/or physically assaulted in the past 12 months (Table 3). Although only 15.2% of participants (135 of 891) reported having low self-esteem, 100% of participants answered yes to at least one question in the stigma and discrimination section of the survey, a series of questions on participants’ emotional/mental health, beliefs of others’ perceptions of transgender identity, and their experience of stigma and discrimination (Table 3 and data not shown).

**Table 3:** Risk Perception, Stigma and Discrimination (S&D), Violence and HIV testing.

	n (%)
**Risk perception of acquiring HIV (n = 785)**	
Very likely/likely	437(55.7)
Unlikely/very unlikely	348 (44.3)
**Experience of discrimination related to TG identity** (n = 891)	
Yes	488 (54.8)
No	403 (45.2)
**Raped and/or physically assaulted in past 12 months** (n = 880)	
No	613 (69.7)
Yes	267 (30.3)
**Have a low self-esteem feeling in the past 12 months** (n = 891)	
No	756 (84.8)
Yes	135 (15.2)
**Previous HIV testing**	
Ever tested for HIV (n = 890)	742 (83.4)
Tested for HIV during the last 12 months (n = 737)	664 (82.8)
Received HIV test results during the last 12 months (n = 643)	613 (72.2)
**Study HIV results** (n = 891)	
HIV negative	854 (95.8)
HIV positive	37 (4.15)
**Location of HIV positive identified during the study**	
Phnom Penh (n = 313)	18 (5.8)
Siem Reap (n = 114)	10 (8.8)
Other locations (n = 464)	9 (2.0)

### HIV testing

The majority of participants had previously tested for HIV (742 of 890, 83.4%), with a higher majority (82.8%) in the past 12 months (Table 3).

Among the 891 participants tested for the study, 37 tested were found HIV positive, representing a 4.15% HIV prevalence rate among the study population. The highest prevalence was found in Siem Reap (8.8%), and in Phnom Penh (5.8%) compared to all other locations (2.0%) (Table 3).

### Logistic Regression to analyze factors associated with HIV positivity among the TG participants

Bivariate analysis found that age, education level, current residence, occupation, receiving payment at first sex, ever use drugs, injecting drugs in the past 12 months, sexual activity during/after using drugs, number of commercial sexual partners during the last 6 months, inconsistent condom use at last anal sex, risk-perception of acquiring HIV, ever being physically assaulted in the past 12 months, and reported low self-esteem were significantly associated with HIV infection among study participants (Table 4). The following variables were not found significantly associated with HIV infection in the univariate analysis: gender identity, relationship/marital status, income, hormone use, age at first sex, type of first sexual partner, sexual partner type (female, male, paying, being paid), inconsistent condom use in the past 6 months, alcohol use in the past 3 months, having anal/vaginal sex after drinking alcohol, ever experienced discrimination, and been raped in the past 12 months (data not shown).

**Table 4:** Logistic Regression Analysis of factors associated with HIV infection among study participants.

(n = 632)			Bivariate analysis		Multivariate Model	
	#	# HIV+ (%)	Crude odds ratios (95% CIs)	p-value	Adjust odds ratios (95% CI)	p- value
**Age at participation into this study**						
<25	378	9 (2.38)	1		1	
25–29	160	10 (6.25)	2.73 (1.09, 6.86)	0.032	2.23 (0.84, 5.95)	0.109
30–34	64	9 (14.06)	6.71 (2.55, 17.63)	<0.001	7.63 (2.55, 22.81)	<0.001
35–44	30	6 (20.00)	10.25 (3.37, 31.19)	<0.001	14.73 (4.20, 51.67)	<0.001
**Education**						
Lower secondary—university & higher	517	18 (3.48)	1		1	
Primary & Never attending school	115	16 (13.91)	4.48 (2.21, 9.09)	<0.001	2.62 (1.18, 5.80)	0.017
**Current residence**						
all other locations	299	7 (2.34)	1		1	
Phnom Penh	244	18 (7.38)	3.32 (1.36, 8.09)	0.008	2.62 (0.94, 7.24)	0.064
Siem Reap	89	9 (10.11)	4.69 (1.69, 12.99)	0.003	7.44 (2.38,23.29)	0.001
**Occupation**						
All other occupations	576	27 (4.69)	1			
Factory worker	56	7 (12.50)	2.90 (1.20, 7.01)	0.018		
**Receiving payment at first sex**						
No	406	16 (3.94)	1		1	
Yes	207	17 (8.21)	2.18(1.08, 4.41)	0.030	2.26 (1.00, 5.11)	0.05
Refuse to answer	19	1 (5.26)	1.35 (0.17, 10.78)	0.775	3.53 (0.39, 32.28)	0.264
**Ever used drugs**						
No	491	20 (4.07)	1			
Yes	141	14 (9.93%)	2.60 (1.28, 5.29)	0.009		
**Inject drugs in past 12 months**						
Never used drugs	491	20 (4.07)	1			
No	75	6 (8.00)	2.048 (0.794, 5.289)	0.138		
Yes	66	8 (12.12)	3.25 (1.37, 7.71)	0.008		
**Sexual activity during/after using drugs** (Heroin and/or Yama and/or Ice)						
No	565	24 (4.25)	1		1	
Yes	67	10 (14.93)	3.95 (1.80, 8.68)	<0.001	2.90 (1.09,7.73)	0.033
**# of commercial sex partners (got paid and/or paid) during last 6 months**						
0	328	12 (3.66)	1			
1–5	139	9 (6.47)	1.82 (0.75, 4.43)	0.185		
>5	165	13 (7.88)	2.25 (1.00, 5.05)	0.050		
**Condom use during the last anal sex with a man**						
Yes	534	24 (4.49)	1		1	
No	98	10 (10.20)	2.41 (1.12, 5.22)	0.025	3.84 (1.58, 9.33)	0.003
**Risk perception of acquiring HIV**						
Likely/Unlikely/Very unlikely	462	9 (1.91)	1			
Very likely	106	8 (8.26)	4.10 (1.55, 10.92)	0.005		
Refuse to answer	31	1 (3.13)	1.62 (0.20, 13.23)	0.651		
**Ever been physically assaulted in past 12 months**						
No	488	21 (4.30)	1			
Yes	144	13 (9.03)	2.21 (1.08, 4.53)	0.031		
**Reported low self-esteem**						
No	532	24 (4.51)	1		1	
Yes	100	10 (10.00)	2.35 (1.09, 5.08)	0.030	3.25 (1.35,7.85)	0.009

Multiple logistic regression analysis (n = 632) was performed to identify factors independently associated with HIV infection. The sample size for this analysis was lower than the original sample size because missing data and the cases in the factors where HIV prevalence was nil (data not shown) were excluded from multivariate analysis. Therefore, HIV prevalence estimates in this multiple regression analysis are different than those bivariate analyses presented above. However, there are no statistically significant differences in the relationships between HIV prevalence and the variable of interest. The final logistic regression model (Table 4) revealed that older age (adjusted OR = 14.73 [4.20, 51.67] for age 35–44 and adjusted OR = 7.63 [2.55, 22.81] for age 30–34), only having a primary school education or no schooling at all (adjusted OR = 2.62 [1.18, 5.80], being a resident of Siem Reap (adjusted OR = 7.44 [2.37,23.29], receiving payment at first sex (adjusted OR = 2.26 [1.00, 5.11], having sex during/after using drugs (adjusted OR = 2.90 [1.09,7.73]), inconsistent condom use during last anal sex (adjusted OR = 3.84 [1.58, 9.33]), and reporting low self-esteem (adjusted OR = 3.25 [1.35,7.85]) were independently associated with HIV infection among study participants.

## Discussion

This study confirms that transgender individuals in Cambodia have a high rate of HIV transmission. The prevalence among participants (4.15%) was higher than that among the general population (0.7%) age 15–49 years [7], as well as higher than the 2010 rate among MSM in the Bros Khmer study: men who have sex with men and women―MSMW (2.2%), and men who have sex with men only―MSMO (2.1%) [6]. These data demonstrate the continued need for HIV prevention and access to care and treatment services for transgender individuals in Cambodia [1, 8].

Although the prevalence among this population is lower than that in other countries with similar epidemic profiles in the Asia region, this study contributes to the understanding of the HIV burden born by transgender individuals. Further, our findings in Cambodia related to the experience and risks facing transgender individuals at the individual, community, and socio-cultural environment levels reflect those highlighted in other studies [1, 2, 4].

Our findings show older participants (≥30 years) have a significantly higher HIV prevalence than those who were younger. As age is likely associated with a longer duration of exposure (cumulative risk over time), it is possible that HIV positive individuals acquired HIV early in Cambodia’s epidemic. These findings suggest that for improving HIV case finding and access to HIV services of transgender individuals in Cambodia, the priority is ensuring older individuals are routinely reached with HIV testing and counseling services.

The HIV prevalence among transgender individuals was significantly higher in Siem Reap province (10.1%) than in Phnom Penh (7.3%)—the country’s capital, or in the other provinces included in this study combined (2.3%). However, combined both cities had a significantly higher prevalence compared with all other areas of study. The reasons for this are unclear; however, it is possible that transgender individuals in urban centers face unique or additional risks compared to other members of this population in the country. For instance, housing Angkor Wat, a world heritage site, Siem Reap is a tourist hotspot. It is possible that the influx of tourists contributes to the practice of riskier sex, including greater frequency of transactional sex. Since we did not collect information about the partner type other than at first sex among our participants, further investigations are needed to understand the specific drivers of increased HIV prevalence in both Siem Reap and Phnom Penh.

Inconsistent condom use during last anal sex was identified as a risk factor for HIV acquisition. Although the majority of sexual activity practiced by participants was with a male partner and involved anal sex, approximately one third of participants reported sex with a female. Consistent condom use in the past six months with both male and female partners, either paid or unpaid, was very limited (44.2%, 243/550). Our study findings also show that consistent condom use with male commercial (43.5%, 171/393) and non-commercial partners (44.9%%, Fig 1B) during the past six months was relatively equal. Yet, consistent condom use with a male partner for purchased sex (20.3%) was far lower than that with non-commercial (44.9%) or with paid partners (48%). The reasons participants have more consistent condom use with non-commercial partners or when being paid for sex than when purchasing sex is unclear. It may be because the bulk of attention has been paid to condom use promotion in transactional sex among transgender individuals when they are the sellers and not the procurers of sex. Additionally, low rates of consistent condom use with non-commercial partners may be explained by transgender individuals having more steady than casual partners that they love and trust than casual partnerships. Further study is needed to investigate this point among transgender individuals in Cambodia.

This condom use pattern could also be related to power dynamics in interpersonal relationships. In heterosexual partnerships, such dynamics indicate that men tend to have greater power than women, and that these power dynamics lead to risky behaviors, such as inconsistent condom use [17–20]. In transgender relationships similar power dynamics exist, and a desire to be perceived as a “real woman,” and/or stigma and discrimination by their partner may increase risk of HIV and other health harms by not using condoms [21–23]. Another aspect of the power imbalance may be the shortage of males desiring relationships with transgender individuals. In response, reducing stigma and discrimination, strengthening transgender individuals’ sexual decision-making power, and ensuring access to quality gender-based violence services are important components of a package of services for transgender individuals. Given the equally low inconsistent condom use rates with commercial and non-commercial partners, our data suggest that condom promotion efforts must strengthen consistent condom use with all partner types among transgender individuals in Cambodia. Although not a comparable time frame, the importance of targeting commercial and non-commercial partnerships among transgender women is evident when examining condom use among female sex workers: consistent condom use with clients in the past three months (80.6%) versus use with sweethearts or steady partners (52.1%)[24].

Although all participants reported having experienced stigma and discrimination, only a portion reported having low self-esteem, which was identified as an independent associated factor for HIV infection among transgender individuals in our study. The determination of poor self-esteem was based on a single question asking interviewees to self-report. It is remarkable that a single question led to the identification of an independent factor for HIV infection among this population. This might give us a way to identify sub-populations of transgender individuals with low self-esteem and at higher risk of HIV infection. However, further study will be needed to validate the reliability of this single question to truly identify poor self-esteem among transgender individuals in Cambodia. These findings also suggest that although some individuals experience stigma and discrimination, they are resilient and able to withstand negative repercussions, while others―those with low self-esteem—are not. Identifying individuals who self-report low self-esteem and understanding what this term means in the Cambodian context is a priority.

Nearly one-third of participants reported having had sex with a female in their lifetime. Of note, transgender women having sex with women was not easily uncovered in the literature, and the “dearth of research on…sex between transgender women and female partners” was identified in a recent systematic review of HIV among transgender women [1]. Our study’s examination of this practice helps to fill this gap. It also highlights that the potential risks from transgender individuals’ sex with females, combined with low consistent condom use, have implications for HIV transmission to the heterosexual population and thus for strategies needed within the HIV response. For instance, the importance of segmenting condom use messages and strategies by partner type, including females, and sex act (i.e. receptive or penetrative, although our study did not explore this practice).

Although the majority of participants did not regularly use drugs, using drugs before or during sex was associated with HIV infection. This association also exists in other settings, where drug use among transgender individuals was used in response to stigma and discrimination, intimate partner violence, and/or physical or mental distress related to sex work [25–28]. Although our study did not examine reasons for drug use, participants did experience similar linked factors. This suggests strategies are needed to minimize drug use among transgender individuals in Cambodia, as well as to address the potential drivers of this practice. Further research to understand reasons and circumstances for drug use is warranted.

Although the present study makes significant contributions to understanding the HIV prevalence and risk behaviors of transgender individuals in Cambodia, there are a number of limitations. To achieve a representative sample, RDS is dependent on the connectivity of networks, yet in Cambodia it is unknown if sub-networks are connected and therefore whether all segments of the transgender population were recruited for this study. In addition, our study was conducted in six cities of Cambodia, and therefore is not representative of the entire country’s transgender population. Our study sample was also well educated, yet lower education was associated with the experience of greater discrimination. It is uncertain if our study sample’s level of education reflects that of all transgender individuals in Cambodia. Another limitation stems from two eligibility criteria. The first is that participants had to have had sex with a male partner in the past 12 months, excluding not recently sexually active transgender individuals and transgender individuals who have had sex only with women. Because this was a study of HIV risk and prevalence, these limitations should not significantly affect the results. The second is that transgender individuals less than 18 years of age were excluded as minors and were not allowed to be included following review by the National Ethics Committee of Cambodia, despite existing global guidance [29]. As a result, this study was unable to quantify the risks young transgender individuals face. This is unfortunate since the majority of participants reported having initiated sex before 18 years, suggesting that transgender individuals less than 18 years are a priority population for HIV prevention. To ensure this population’s needs are understood and addressed, there appears to be a need to disseminate guidance on research with minors among national ethical committees to allow specific research targeting young at-risk populations.

Despite these limitations, the present integrated biological and behavioral surveillance study among transgender individuals in Cambodia confirms that transgender individuals have a high rate of HIV infection, with older age (>35 years), residence in Siem Reap, lower education, having been paid at first sex, having sex during/after drug use, not using a condom during last anal sex, and low perceived self-esteem being independently associated with HIV infection. Following the results of this study, and in keeping with research-to-practice principles and NCHADS’ boosted CoPCT SOP, a new branded peer outreach and community-based HIV testing program, named *Srey Sros*, for transgender individuals was developed and piloted in Siem Reap where the transgender individual HIV prevalence was found to be higher. This program delivers a comprehensive package of services that address key risk behaviors and associated factors identified in this study that both directly and indirectly contribute to HIV transmission. Recognizing the multiple levels of risk transgender individuals’ face, Srey Sros addresses these risks at the individual, community and structural levels, for instance by working to improve self-esteem and to reduce stigma and discrimination. A careful evaluation of this program is planned to further inform this program and improve the effectiveness of the HIV response among transgender individuals in Cambodia. For instance, understanding the reasons transgender individuals in Siem Reap province have a higher HIV prevalence than in other parts of the country might have important programmatic implications. The next IBBS survey, planned for 2015, will also provide further important insights. Finally, given the high rates of HIV prevalence and high-risk behaviors among transgender individuals in Cambodia, pre-exposure prophylaxis could represent an important intervention to reduce HIV transmission among this population [30].

## Supporting Information

S1 Dataset (DTA)

S1 Questionnaire (DOCX)

## References

[1] Baral S. D., Poteat T., Strömdahl S., Wirtz A. L., Guadamuz T. E., Beyrer C. (2013). Worldwide burden of HIV in transgender women: a systematic review and meta-analysis. *The Lancet infectious diseases*, 13(3), 214–222. 10.1016/S1473-3099(12)70315-8 23260128

[2] Herbst J. H., Jacobs E. D., Finlayson T. J., McKleroy V. S., Neumann M. S., Crepaz N., et al (2008). Estimating HIV prevalence and risk behaviors of transgender persons in the United States: a systematic review. *AIDS and Behavior*, 12(1), 1–17. 17694429 10.1007/s10461-007-9299-3

[3] Operario D., Soma T., Underhill K. (2008). Sex work and HIV status among transgender women: systematic review and meta-analysis. *JAIDS Journal of Acquired Immune Deficiency Syndromes*, 48(1), 97–103. 10.1097/QAI.0b013e31816e3971 18344875

[4] Poteat T., Wirtz A. L., Radix A., Borquez A., Silva-Santisteban A., Deutsch M. B., et al (2015). HIV risk and preventive interventions in transgender women sex workers. *The Lancet*, 385(9964), 274–286. 10.1016/S0140-6736(14)60833-3 PMC4320978 25059941

[5] Sopheab H., Morineau G., Neal J. J., Chhorvann C. (2005). Cambodia STI Prevalence Survey, Integrated Biological and behavioural Survey: Sexually transmitted infections and related behaviours among brothel-based female sex-workers, police, and men who have sex with men 2008. Phnom Penh, Cambodia: NCHADS.

[6] Liu K. L., Chhea C. (2010). The BROS Khmer: Behavioral risks on-site serosurvey among at-risk urban men in Cambodia. Phnom Penh, Cambodia: FHI, 360.

[7] Chhorvann C., Liu K. L. (2007). Behavioral Surveillance Survey: HIV/AIDS Related Sexual Behaviors among Sentinel Groups. Phnom Penh, Cambodia: NCHADS.

[8] World Health Organization. (2011). Prevention and treatment of HIV and other sexually transmitted infections among men who have sex with men and transgender people: recommendations for a public health approach. Geneva: WHO. 26158187

[9] National Center for HIV/AIDS, Dermatology & STIs (NCHADS). (2013). Boosted continuum from prevention to care and treatment. Phnom Penh, Cambodia: NCHADS.

[10] Heckathorn D. D. (1997). Respondent-driven sampling: a new approach to the study of hidden populations. *Social problems*, 44(2):174–199.

[11] Joint United Nations Programme on HIV/AIDS. (2011). UNAIDS terminology guidelines. Geneva: Joint United Nations Programme on HIV/AIDS.

[12] National Center for HIV/AIDS, Dermatology & STIs (NCHADS). (2006). Serologic assays for Human Immunodeficiency virus Antibody in Dry Blood Specimens Collected on Filter Paper. Phnom Penh, Cambodia: NCHADS.

[13] Girault P., Saidel T., Song N., Van Wijngaarden J. W. D. L., Dallabetta G., Stuer F., et al (2004). HIV, STIs, and sexual behaviors among men who have sex with men in Phnom Penh, Cambodia. *AIDS Education and Prevention*, 16(1: Special issue), 31. 15058709 10.1521/aeap.16.1.31.27727

[14] UNDP, USAID. (2014). Being LGBT in Asia: Thailand Country Report. Bangkok.

[15] FHI 360. (2000). Behavioral Surveillance Surveys (BSS), in Guideline for Repeated behavioral Surveys in Population at Risk of HIV. Washington DC: USA: FHI 360.

[16] IPPF. (2008). People Living with HIV Stigma Index: User Guide. London, UK: IPPF.

[17] Felmlee D. H. (1994). Who's on top? Power in romantic relationships. *Sex Roles*, 31(5–6), 275–295.

[18] Pulerwitz J., Amaro H., Jong W. D., Gortmaker S. L., Rudd R. (2002). Relationship power, condom use and HIV risk among women in the USA.*AIDS care*, 14(6), 789–800. 12511212 10.1080/0954012021000031868

[19] Harvey S. M., Bird S. T., Galavotti C., Duncan E. A., Greenberg D. (2002). Relationship power, sexual decision making and condom use among women at risk for HIV/STDs. *Women & Health*, 36(4), 69–84. 12555803 10.1300/J013v36n04_06

[20] Pettifor A. E., Measham D. M., Rees H. V., Padian N. S. (2004). Sexual power and HIV risk, South Africa. *Emerging infectious diseases*,10(11), 1996–2004. 15550214 10.3201/eid1011.040252 PMC3328992

[21] Lombardi E. L., Wilchins R. A., Priesing D., Malouf D. (2002). Gender violence: Transgender experiences with violence and discrimination. *Journal of homosexuality*, 42(1), 89–101. 10.1300/j082v42n01_05 11991568

[22] Melendez R. M., Pinto R. (2007). ‘It's really a hard life’: Love, gender and HIV risk among male-to-female transgender persons. *Culture*, *health & sexuality*, 9(3), 233–245. 10.1080/13691050601065909 PMC3539165 17457728

[23] Operario D., Nemoto T., Iwamoto M., Moore T. (2011). Unprotected sexual behavior and HIV risk in the context of primary partnerships for transgender women. *AIDS and Behavior*, 15(3), 674–682. 10.1007/s10461-010-9795-8 20740376 PMC3049202

[24] National Center for HIV/AIDS, Dermatology & STIs (NCHADS). (2013). Biological Behavioral Surveillance Study (BSS). Phnom Penh, Cambodia: NCHADS.

[25] Reback C. J., Fletcher J. B. (2014). HIV prevalence, substance use, and sexual risk behaviors among transgender women recruited through outreach.*AIDS and Behavior*, 18(7), 1359–1367. 10.1007/s10461-013-0657-z 24287786 PMC4209944

[26] Sevelius J. M., Reznick O. G., Hart S. L., Schwarcz S. (2009). Informing interventions: the importance of contextual factors in the prediction of sexual risk behaviors among transgender women. *AIDS education and prevention*: *official publication of the International Society for AIDS Education*, 21(2), 113. 19397434 10.1521/aeap.2009.21.2.113 PMC4535696

[27] Hotton A. L., Garofalo R., Kuhns L. M., & Johnson A. K. (2013). Substance use as a mediator of the relationship between life stress and sexual risk among young transgender women. *AIDS Education and Prevention*, 25(1), 62 10.1521/aeap.2013.25.1.62 23387952

[28] Keuroghlian A. S., Reisner S. L., White J. M., Weiss R. D. (2015). Substance use and treatment of substance use disorders in a community sample of transgender adults. *Drug and alcohol dependence*, 152, 139–146. 10.1016/j.drugalcdep.2015.04.008 25953644 PMC4458188

[29] Schenk K., Williamson J. (2005). Ethical approaches to gathering information from children and adolescents in international settings: guidelines and resources. Washington, DC: Population Council.

[30] World Health Organization. (2014). Consolidated guidelines on HIV prevention, diagnosis, treatment and care for key populations. Geneva: WHO. 25996019

