# Supplementary material for: HIV Prevalence and Risks Associated with HIV Infection among Transgender Individuals in Cambodia
Source: PLoS One. 2016 Apr 12;11(4):e0152906. doi: 10.1371/journal.pone.0152906 (PMC4829243; doi:10.1371/journal.pone.0152906)
Supplement: S1 Questionnaire — (DOCX) [file pone.0152906.s002.docx]

Participant ID Code: ..................................

***FHI 360 & National Center for HIV/AIDS Dermatology and STD (NCHADS)***

**Integrated Biological and Behavioral Survey of Transgender Population in Cambodia, 2012**

*Revised 15/6/2012*

**To be entered in the computer by a research team member before the interview:**

Date of interview: .day...............month..................year...............

Province/City:

1- Phnom Penh

2- Banteay Meanchey – Serey Sophorn

3- Banteay Meanchey – Poipet

4- Battambang

5- Kampong Cham

6- Siem Reap

7- Preah Sihanouk

Interview Location (City/NGOs):…………………………………………

Who conducts the interview?

1- Self-administered

2- Interviewer-administered

**Introduction:**  Paragraph below is to be listened by the participant via audio headsets

**Introduction:** (The following is in audio recording format): "FHI 360 & NCHADS are conducting a survey of transgender people in Cambodia to learn more about their HIV knowledge and risk. We would like to request your cooperation for about 30 minutes to ask you a few questions. Some of these questions are personal. You are free to refuse to give the answers at any time. All answers are totally confidential. You do not need to reveal your name and there is no way that anyone can identify how you answered these questions. Please be totally truthful in your responses. Your participation is very important and will help Cambodia to improve its health services for people like you. May we start the interview now?"

| **SECTION 1: SOCIODEMOGRAPHIC CHARACTERISTICS** | | | |
| --- | --- | --- | --- |
| **No.** | **Questions and filters** | **Coding categories** | **Skip to** |
| **Now I would like to ask you some questions related to your personal information.** | | | |
| Q101 | How old are you? | Number of years:......................................  (in complete years) |  |
| Q102 | Are you currently married?  **(only one response)** | Yes, married and living together 1  Yes, married but not living together 2  Widowed, Divorced or separated 3  Not married and not living with any partner 4  Not married but living with sweetheart (female) 5  Not married but living with male lover 6 |  |
| Q103 | Which province were you born? | Phnom Penh 1  Kandal 2  Banteay Meanchey 3  Battambang 4  Siem Reap 5  Kampong Cham 6  Preah Sihanouk 7  Kampong Speu 8  Kampot 9  Kampong Chhnang 10  Koh Kong 11  Kratie 12  Prey Veng 13  Kampong Thom 14  Pursat 15  Ratanakiri 16  Oddar Meanchey 17  Svay Rieng 18  Stung Treng 19  Pailin 20  Takeo 21  Preah Vihear 22  Mondulkiri 23  Kep 24 |  |
| Q104 | Which province/city is your primary residence? | Phnom Penh 1  Kandal 2  Banteay Meanchey 3  Battambang 4  Siem Reap 5  Kampong Cham 6  Preah Sihanouk 7  Kampong Speu 8  Kampot 9  Kampong Chhnang 10  Koh Kong 11  Kratie 12  Prey Veng 13  Kampong Thom 14  Pursat 15  Ratanakiri 16  Oddar Meanchey 17  Svay Rieng 18  Stung Treng 19  Pailin 20  Takeo 21  Preah Vihear 22  Mondulkiri 23  Kep 24 |  |
| Q105 | For how long have you been living in this city? | .......Years........Months  **Recode 0 if** doesn’t live in this city  All my life 88  Refuse to answer 99 |  |
| Q106 | How much money do you usually make every month? | Amount of money (in Riel): …………….  Refuse to answer 99 |  |
| Q107 | Does your income meet with your expenditure? | Yes 1  No 0  Refuse to answer 99 |  |
| Q108 | How many years did you complete at school? | Number of year: .......................................................  **Recode 0 if** never attending school.  Refuse to answer 99 |  |
| Q109 | What is your current job (main source of income)?  **(only one response)**  *Note: If you are both studying and having a paid job, report your main source of income.* | Unemployed 0  (Moto/tuktuk Taxi/truck/private) Driver 1  Teacher 2  Factory Worker 3  Farmer/Fisherman 4  Store Seller 5  Street Vendor 6  Office Worker 7  Restaurant/Cafe worker (Waiter/Cook/Bartender) 8  Sex Worker 9  Student 10  Hair dresser/Beautician 11  NGO staff 12  Government Officer 13  Other 89 |  |

| **Section 2. HIV PREVENTION PROGRAMS** | | | | | |
| --- | --- | --- | --- | --- | --- |
| **No.** | **Questions and filters** | | **Coding categories** | | **Skip to** |
| Q201 | Did you participate in the BROS Khmer study with HIV rapid testing and computerized survey in 2010?  *[Show BROS Khmer logo]* | Yes 1  No 0  Refuse to answer 99 | |  | |
| Q202 | In the **past 3 months**, did you receive any education and/or information which related to HIV/AIDS, social and health risks? | Yes 1  No 0  Refuse to answer 99 | | **0→ Q204** | |
| Q203 | Through what method have you received this education and/or information in the past 3 months?  [Multiple Answers] | TV 1  Radio 2  Newspaper 3  Billboard 4  Poster/leaflet 5  Booklet 6  Lecture/Training 7  Focus group discussion 8  Talking to outreach worker/peer educator 9  Hospital/Clinic/Health Center 10  Other 89 | |  | |
| Q204 | Where can you get condoms for free?  [Multiple Answers] | Never got condoms 1  At a brothel/entertainment venue 2  At a drug store/pharmacy 3  At vender on street 4  Peer Educator/NGOs 5  Health Center/Clinic 6  Not remember 7  Other place 8 | |  | |
| Q205 | Have you ever heard of MStyle Program?  *[Show MStyle logo]* | Yes 1  No 0  Refuse to answer 99 | | **0→ Q301**  **99→ Q301** | |
| Q206 | Through what method have you learned about MStyle program?  [Multiple Answers] | TV 1  Radio 2  Newspaper 3  Billboard 4  Poster 5  Booklet 6  Lecture/Training 7  Focus group discussion 8  Talking to outreach worker 9  Hospital/Clinic/Health Center 10  MStyle Web site 11  Other sites on Internet (e.g., Facebook) 12  Other 89 | |  | |

| **Section 3. TRANSGENDER IDENTITY & EXPERIENCES** | | | |
| --- | --- | --- | --- |
| Q301 | What gender are you identified with? | Female 1  Male 2  Third sex 3 |  |
| Q302 | How often do you express yourself as a woman, such as dressed or acted like a woman? | Sometimes 1  All the time 2  Never 3 |  |
| Q303 | Generally, do you keep long hair or short hair? | Long hair 1  Short hair 2  Both 3 |  |
| Q304 | How many friends in your social network are also transgender? | Number: ............................... | **→ Number needs to be greater than 0** |
| Q305 | Have you ever injected female hormone or/and taken hormone pills? | Yes 1  No 0  Refuse to answer 9999 | **0→ Q310** |
| Q306 | What is the primary method you use(d) to take female hormones? | Pills 1  Injections 2  Skin patches 3  Other 4  Refuse to answer 98 |  |
| Q307 | How long have you been taking female hormone? | ______Years _______Months  Refuse to answer 99 |  |
| Q308 | Have you ever stopped taking female hormone? | Yes 1  No 0  Refuse to answer 99 | **0→ Q310** |
| Q309 | Why? | because of the expense 1  because of unwanted side effects 2  because I became concerned about long-term health risks 3  because they didn’t work 4  Other 5  Refuse to answer 99 |  |
| Q310 | What feminization procedures have you undertaken?  (Multiple answers) | Breast implants (injections) 1  Breast implants (not injections) 2  Removal of the penis 3  Creation of a vagina 4  Implants (not injections) in any other part of the body (buttocks, hips, etc.)5  Voice feminization surgery 6  Facial feminization surgery (chin, jawline, forehead, etc.) 7  Refuse to answer 99 | **0→ Q313** |
| Q311 | What was your main source to get the money to pay for medical surgery to change your sex? | Employed 1  Self-employed 2  Selling sex 3  My family/relatives 4  Friends 5  Boss/Employer 6  My sweetheart/boyfriend 7  Other 9  Refuse to answer 99 |  |
| Q312 | Are you satisfied with that surgery? | Very satisfied 1  Somewhat satisfied 2  Somewhat unsatisfied 3  Very unsatisfied 4  Refuse to answer 99 | **All Skip to Q314** |
| Q313 | So, you have not got the sex change procedures, how likely are you going to get it? | Very Likely 1  Likely 2  Unlikely 3  Very Unlikely 4  Refuse to answer 99 |  |
| Q314 | What feminization procedures would you like to undertaken in the future?  (Multiple answers) | Breast implants (injections) 1  Breast implants (not injections) 2  Removal of the penis 3  Creation of a vagina 4  Implants (not injections) in any other part of the body (buttocks, hips, etc.)5  Voice feminization surgery 6  Facial feminization surgery (chin, jawline, forehead, etc.) 7  Refuse to answer 99 |  |
| Q315 | How many **persons like you** do you estimate in this province? | Number: ...............................  Recode 0 if did not know anyone  Refuse to answer 998 |  |
| Q316 | Among these people, how many with **sex-change surgery** do you estimate in this province? | Number: ...............................  Recode 0 if did not know anyone  Refuse to answer 998 |  |

| **Section 4. STI AND HIV TESTING** | | | | | |
| --- | --- | --- | --- | --- | --- |
| **No.** | **Questions and filters** | | **Coding categories** | | **Skip to** |
| Q401 | In the past 12 months, have you experienced the following symptoms?   \| **No.** \| **symptoms** \| **Yes** \| **No** \| Refuse to answer \| \| --- \| --- \| --- \| --- \| --- \| \| a \| Cuts or sores in the genital area \| **1** \| **0** \| **99** \| \| b \| Swelling in the genital area \| **1** \| **0** \| **99** \| \| c \| Urethral discharge with an unpleasant smell \| **1** \| **0** \| **99** \| \| d \| Crab (lice) \| **1** \| **0** \| **99** \| | | | | |
| Q402 | Where did you first go for treatment the last time you had any symptom above?  (only one response) | Never had any symptom 0  Pharmacy 1  Private clinic 2  Public Hospital/STD clinic 3  Clinic NGO 4  Traditional doctor 5  Didn’t get care 6  Other 89 | |  | |
| Q403 | Have you ever been tested for HIV before? | Yes 1  No 0  Refuse to answer 99 | | **0→ Q407** | |
| Q404 | Have you been tested for HIV in past 12 months? | Yes 1  No 0  Refuse to answer 99 | | **0→ Q407** | |
| Q405 | Last time when within the last 12 months, you got tested, did you receive the result of the test you took? | Yes 1  No 0  Refuse to answer 99 | |  | |
| Q406 | Where did you have your last HIV test in the past 12 months? | Private lab or clinic 1  Public hospital 2  VCT/NGO-CIPCT 3  HIV sentinel surveillance or other studies 4  Other 89 | |  | |
| Q407 | What is your current HIV status?  (You could choose not to answer by checking ‘9’) | Positive 1  Negative 2  I don’t know my status 3  I am not telling you 9 | | **1→ Q501** | |
| Q408 | Do you feel yourself are at risk to be infected with HIV? | Very Likely 1  Likely 2  Unlikely 3  Very Unlikely 4  Refuse to answer 99 | |  | |

| **Section 5. SEXUAL PARTNERS AND SEXUAL HISTORY** | | | | | | |
| --- | --- | --- | --- | --- | --- | --- |
| **No.** | | **Questions and filters** | | **Coding categories** | | **Skip to** |
|  | | **Now I would like to ask some questions about your recent sexual relationship with various kinds of sexual partners.** | | | |  |
| Q501 | How old were you when you had sexual intercourse (, anal, or vaginal) for the first time? | | Age in years:............................ | |  | |
| Q502 | Was your first sexual partner a man, a woman or transgender? | | Man 1  Woman 2  Transgender 3  Refuse to answer 99 | |  | |
| Q503 | Who was your first sexual partner? | | sweetheart/boyfriend/girlfriend 1  spouse 2  friend 3  stranger 4  Family/relatives 5  Refuse to answer 99 | |  | |
| Q504 | Did you get paid for the first sex? | | Yes 1  No 2  Refuse to answer 99 | |  | |
| Q505 | Did you pay for the first sex? | | Yes 1  No 2  Refuse to answer 99 | |  | |
| **SEX WITH WOMEN** | | | | | | |
| Q601 | Have you ever had sex with a woman? **(Including vaginal, or anal sex)** | | Yes 1  No 0 | | **0→ Q701** | |
| Q602 | In the **past 6 months**, did you have sex with a woman? [Including vaginal, or anal sex] | | Yes 1  No 0 | | **0→ Q701** | |
| Q603 | In **the past 6 months**, how often did you use a condom when you had vaginal or anal sex with women without pay? | | Always 1  Often 2  Sometimes 3  Never 4  Refuse to answer 99 | | **1→ Q605a** | |
| Q604 | **In the past 6 months** what **are the reasons** that you did not use a condom in vaginal or anal sex with a woman without pay?  **[Multiple Answers]** | | We are in a relationship 1  She is not HIV/STI infected 2  Too high to use a condom 3  No condom available 4  Feel better without a condom 5  I never like condoms 6  I am HIV-infected 7  I penetrated her, so I am not at risk 8  I trust her or she trusts me 9  Other 89  Refuse to answer 99 | |  | |
| Q605a | In **the past 6months, women** have you **paid** any woman to have sex with? | | Yes 1  No 0 | | **0→ Q609** | |
| Q605 | In **the past 6months, how many women** have you **paid** to have sex with?  (Buying sex means you pay money to sexual partner) | | Number of women: ...............................  **Record 0** if did not buy sex **then Skip to Q609**    Refuse to answer 99 | | **0→ Q609** | |
| Q606 | In the past 6 months where did you **meet** your women you paid for sex? | | Park 1  Street 2  Bar/discotheque/cafe 3  Beer Garden/Restaurant 4  Massage Parlor 5  Karaoke 6  Suspected brothels 7  Other 89 | |  | |
| Q607 | In **the past 6 months**, how often did you use a condom when you had vaginal or anal sex with a woman who you paid? | | Always 1  Often 2  Sometimes 3  Never 4  refuse to answer 99 | |  | |
| Q608 | **In the past 6 months**, what is the **main reason** that you did not use a condom in vaginal or anal sex with a woman you paid last time?  [Including vaginal, or anal sex]  **(only one response)** | | We are in a relationship 1  She is not HIV/STI infected 2  I was drinking or using drug (too high) 3  No condom available 4  Feel better without a condom 5  I never like condoms 6  I am HIV-infected 7  I penetrated her, so I am not at risk 8  I trust her or she trusts me 9  Other 89  Refuse to answer 99 | |  | |
| Q609 | In **the past 6 months,** did any **women** pay you money/gifts for sex? | | Yes 1  No 0  Refuse to answer 99 | | **0→ Q701** | |
| Q610 | Where did you **meet** your female clients who paid you money to have sex in past 6 months?  **[Multiple Answers]** | | Park 1  Street 2  Bar/discotheque/cafe 3  Beer Garden/Restaurant 4  Massage Parlor 5  Karaoke 6  Hotel 7  Gym 8  Party 9  Other 89 | |  | |
| Q611 | In **the past 6 months,**, with all of these female clients paying you for sex, how often did you **use** a **condom**? | | Always 1  Often 2  Sometimes 3  Never 4  Refuse to answer 99 | |  | |
| Q612 | What are the **reasons** that you did not use a condom in vaginal or anal sex with a female client who paid you money to have sex in last 6 months?  [Including vaginal, or anal sex]  **[Multiple Answers]** | | We are in a relationship 1  She is not HIV/STI infected 2  Too high to use a condom 3  No condom available 4  Feel better without a condom 5  I never like condoms 6  I am HIV-infected 7  I penetrated her, so I am not at risk 8  I trust her or she trusts me 9  Other 89  Refuse to answer 99 | |  | |

| **SEX WITH MEN** | | | |
| --- | --- | --- | --- |
| Q701 | Have you ever had anal sex with a man? | Yes 1  No 0 | **0→ Q801** |
| Q702 | In the **past 6 months**, did you have anal sex with a man? | Yes 1  No 0 | **0→ Q716** |
| Q703 | In the **past 6 months**, what was your usual role in anal sex with a man? | Insertive 1  Receptive 2  Both 3  Refuse to answer 99 |  |
| Q704 | In the **past 6 months**, did you have anal sex with more than 1 man at the same time (i.e., group sex, orgy)? | Yes 1  No 0  Refuse to answer 99 | **0→ Q706** |
| Q705 | In the past 6 months in this group sex, did you use condoms in any **anal sex with men in the group**? | No anal sex 0  Yes 1  No 2  Refuse to answer 99 |  |
| Q706 | In **the past 6 months**, how often did you **use** **a condom** when you had **anal sex** with a man without pay? | No anal sex 0  Always 1  Often 2  Sometimes 3  Never 4  Refuse to answer 99 | **If ‘0’, ‘1’ → Q712a** |
| Q707 | What are the **reasons** that you or your partner did not use a condom in the anal sex with a man without pay in past 6 months?  **[Multiple Answers]** | We are in a relationship 1  He is not HIV/STI infected 2  Too high to use a condom 3  No condom available 4  Feel better without a condom 5  I never like condoms 6  I am HIV-infected 7  I penetrated him, so I am not at risk 8  I trust him or he trusts me 9  Other 89  Refuse to answer 99 |  |
| Q708a | In **the past 6 months,** have **you paid any man** to have anal sex with you? | Yes 1  No 0 | **0→ Q712a** |
| Q708 | In **the past 6 months,** how many men have **you paid** to have anal sex with you?  (paid : you pay money for sex from sexual partner) | Number of men: ....................................  **Record 0** if did not buy sex from men **then skip to Q712**    Refuse to answer 99 | **0→ Q712a** |
| Q709 | **In the past 6 months** where did you **meet** your male partners you paid for anal sex?  **[Multiple Answers]** | Park 1  Street 2  Bar/disco/cafe 3  Friends (social network) 4  Internet 5  Massage parlor/Sauna 6  Other 89 |  |
| Q710 | In **the past 6 months,** how often did you **use** a **condom** when you had **anal sex** when buying sex from a man? | No anal sex 0  Always 1  Often 2  Sometimes 3  Never 4  Refuse to answer 99 | **If ‘0’, ‘1’ → Q712a** |
| Q711 | What are the **reasons** that you or your partner did not use a condom in the anal sex with a man **you paid** in past 6 months?  **[Multiple Answers]** | We are in a relationship 1  He is not HIV/STI infected 2  Too high to use a condom 3  No condom available 4  Feel better without a condom 5  I never like condoms 6  I am HIV-infected 7  I penetrated him, so I am not at risk 8  I trust him or he trusts me 9  Other 89  Refuse to answer 99 |  |
| Q712a | In the **past 6 months**, **have any** male **client** paid you for sex? | Yes 1  No 0 | **0→ Q716** |
| Q712 | In the **past 6 months**, **how many** male **clients** have paid you for sex? | Number of male clients: ...............................  **Record 0** if did not have any client in the past month **then Skip to Q716**  Refuse to answer 99 |  |
| Q713 | **In the past 6 months** where did you **meet** your male clients?  **[Multiple Answers]** | Park 1  Street 2  Bar/disco/cafe 3  Friends (social network) 4  Internet 5  Massage parlor/Sauna 6  Other 89 |  |
| Q714 | In the **past 6 months,** how often did you **use** a **condom** when you had **anal** **sex** with those **male clients**? | No anal sex 0  Always 1  Often 2  Sometimes 3  Never 4  Refuse to answer 99 | **If ‘0’, ‘1’ → Q716** |
| Q715 | What are the **reasons** that you or your partner did not use a condom in the anal sex with a **male client** in past 6 months?  **[Multiple Answers]** | We are in a relationship 1  He is not HIV/STI infected 2  Too high to use a condom 3  No condom available 4  Feel better without a condom 5  I never like condoms 6  I am HIV-infected 7  I penetrated him, so I am not at risk 8  I trust him or he trusts me 9  Other 89  Refuse to answer 99 |  |
| Q716 | How many months ago since you had the last anal sex with a man? | Number of months…………………..  (if less than one month , please record 1) |  |
| Q717 | In the last anal sex, what was your role? | Insertive 1  Receptive 2  Both 3  Refuse to answer 99 |  |
| Q718 | Did you use condom in the last anal sex with a man? | Yes 1  No 0 |  |

| **Section 6. ALCOHOL AND DRUG USE** | | | | | | |
| --- | --- | --- | --- | --- | --- | --- |
| **No.** | | **Questions and filters** | **Coding categories** | **Skip to** | |  |
| Q801 | In the **past 3 months**, how often did you drink alcohol? | | Never 1  Daily 2  weekly 3  At least once 4  Refuse to answer 99 | | 1→ **Q803** | |
| Q802 | In the **past 3 months**, did you have anal or vaginal sex after/during having drunk alcohol? | | Yes 1  No 0  Refuse to answer 99 | |  | |
| Q803 | Some people tried different drugs for different reasons (such as for recreational activities, energy boosting for working longer etc). Have you ever used any drug before? | | Yes 1  No 0  Refuse to answer 99 | | **0→ Q901** | |
| Q804 | In the **past 12 months** have you ever had injected any drug? | | Yes 1  No 0  Refuse to answer 99 | |  | |
| Q805 | Have you tried any of the following drugs in the **past 12 months** ? (Ask one by one – CIRCLE YES OR NO)   \|  \| **Types of drug** \| **Yes** \| **No** \| \| --- \| --- \| --- \| --- \| \| **1** \| Marijuana \| **1** \| **0** \| \| **2** \| Heroin \| **1** \| **0** \| \| **3** \| Yama (amphetamine) \| **1** \| **0** \| \| **4** \| Crystal, Ice (Methamphetamine) \| **1** \| **0** \| \| **5** \| Ecstasy \| **1** \| **0** \| \| **6** \| Opium \| **1** \| **0** \| \| **7** \| Inhalants (glue, paint, petrol, spray can) \| **1** \| **0** \| \| **8** \| Other \| **1** \| **0** \| | | | | | |
| Q806 | How often did you use drugs in the past 3 months?  Heroin  Yama  Ice/Crystalhese in the past three months with a man? | | **Never Daily weekly At least once**    0 1 2 3  0 1 2 3  0 1 2 3 |  | | |
| Q807 | Did you inject these drugs in the past 3 months?  Heroin  Yama  Ice, Amphetamine | | **No Yes**    0 1  0 1  0 1 |  | | |
| Q808 | Did you have sex during/after using these drugs in the past 3 months?  Heroin  Yama  Ice, Amphetamine | | **No Yes**    0 1  0 1  0 1 |  | | |

| **Section 7. ADDITIONAL PREVENTION KNOWLEDGE** | | | |
| --- | --- | --- | --- |
| **No.** | **Questions and filters** | **Coding categories** | **Skip to** |
| Q901 | Can the risk of HIV transmission be reduced by having sex with only one uninfected partner who has no other partners? | Yes 1  No 0 |  |
| Q902 | Can a person reduce the risk of getting HIV by using a condom every time they have sex? | Yes 1  No 0 |  |
| Q903 | Can a healthy-looking person have HIV? | Yes 1  No 0 |  |
| Q904 | Can a person get HIV from mosquito bites? | Yes 1  No 0 |  |
| Q905 | Can a person get HIV by sharing food with someone who is infected? | Yes 1  No 0 |  |
| Q906 | Do you know where you can go if you wish to receive an HIV test? | Yes 1  No 0 |  |
| Q907 | In the past 12 months, have you been given condoms? (e.g., through an outreach service, drop-in center or sexual health clinic) | Yes 1  No 0 |  |

| **Section 8. Stigma and Support** | | | |
| --- | --- | --- | --- |
| **No.** | **Questions and filters** | **Coding categories** | **Skip to** |
| Q1001 | How do your family members (parents, siblings) react to your transgender identity?  [Multiple Answers] | They don’t know at all 0  They support my identity 1  They ignore me 2  They kicked me out of the family 3  They criticize/blame/verbal abuse me 4  They conduct violence/physical abuse on me 5  They put more work for me 6  They lock /restrict me from going outside 7  Other 9  Refuse to answer 99 |  |
| Q1002 | How do non-transgender friends react to your transgender identity?  [Multiple Answers] | They don’t know at all 0  They support my identity 1  They ignore / refused to talk me 2  They kicked me out of the group 3  They criticize/blame/verbal abuse me 4  They conduct violence/physical abuse on me 5  They gossip about me 6  Other 9  Refuse to answer 99 |  |
| Q1003 | How do your employer or co-workers react to your transgender identity?  [Multiple Answers] | They don’t know at all 0  They support my identity 1  They ignore / refused to talk me 2  They criticize/blame/verbal abuse me 3  They conduct violence/physical abuse on me 4  They gossip about me 5  They fire me from work 6    Other 9  Refuse to answer 99 |  |
| Q1004 | In the last 12 months, have you experienced any of the following feelings because of your transgender identity?  [Multiple answers] | I feel ashamed 1  I feel guilty 2  I blame myself 3  I blame others 4  I have low self-esteem 5  I feel I should be punished 6  I feel suicidal 7 |  |
| Q1005 | In the last 12 months, have you done any of the following things because of your transgender identity?  [Multiple answers] | I have chosen not to attend social gathering 1  I have isolated myself from my family and/or friends 2  I decided to stop working 3  I decided not to apply for a job or for a promotion 4  I withdrew from education/training 5  I decided not to get married 6  I decided not to have sex 7  I decided not to have children 8  I avoided going to a local clinic when I needed to 9  I avoided going to a hospital when I needed to 10 |  |
| Q1006 | In the last 12 months, have you been fearful of any of the following things happening to you – whether or not they actually have happened to you?  [Multiple answers] | Being gossiped about 1  Being verbally insulted, harassed or threatened 2  Being physically harassed or threatened 3  Being physically assaulted 4  Refuse to answer 99 |  |
| Q1007 | In the past 12 months, have you ever been raped or forced to engage in sexual contact when you didn’t want to? | Yes 1  No 0  Refuse to answer 99 | **0→ Q1009** |
| Q1008 | Who was responsible for forcing you to engage in sexual contact in the past 12 months?  [Multiple Answers] | Male spouse or sweetheart 1  Female spouse or sweetheart 2  Casual unpaid partner 3  Friend 4  Family member 5  Client 6  Employer 7  Police 8  Gangster 9  Other 10  Refuse to answer 99 |  |
| Q1009 | In the past 12 months, have you ever been physically assaulted by someone who intended to hurt you? | Yes 1  No 0  Refuse to answer 99 | **0→ Q1011** |
| Q1010 | Who was responsible for physically assaulting you during the last 12 months?  [Multiple Answers] | Male spouse or sweetheart 1  Female spouse or sweetheart 2  Casual unpaid partner 3  Friend 4  Family member 5  Client 6  Employer 7  Police 8  Gangster 9  Other 10  Refuse to answer 99 |  |
| Q1011 | When you have emotional problems in your life, whom do you go for help?  [Multiple Answers] | Nobody helps me 0  Friends 1  Family/Relatives 2  Sweethearts/boyfriend/girlfriend 3  Co-workers 4  NGO program staff 5  Health care providers 6  Other 9  Refuse to answer 99 |  |
| Q1012 | For the people like you, what specific HIV prevention, care and treatment program are needed?  [Multiple Answers] | Less harassment from police 1  Protection by laws 2  Public awareness to accept transgender 3  Social support groups for transgender 4  Special health care for transgender 5  Transgender NGO/network 6  More NGO programs for transgender 7  Livelihood support to transgender (microfinance/ job training) 8  Other 9  Refuse to answer 99 |  |

| **Section 9. ACASI Format** | | | | |
| --- | --- | --- | --- | --- |
| **No.** | **Questions and filters** | **Coding categories** | | **Skip to** |
| Q1101 | So far, is it easy or difficult for you to answer these questions on the touch screen of this iPad or on the laptop computer? | Very Difficult 1  Difficult 2  Easy 3  Very Easy 4  Refuse to answer 99 |  | |
| Q1102 | Compared to face-to-face interview, do you think that using computer to answer questions would make yourself more comfortable to answer some sensitive questions (such as sex or drinking)? | Very Likely 1  Likely 2  Unlikely 3  Very Unlikely 4  Refuse to answer 99 |  | |

**[**"Thank you very much for answering these questions. Let us repeat that your answers are totally confidential and there is no way anyone will learn what you told us.**]**
